# Supplementary material for: Liquid Chromatography Coupled to High-Resolution Mass Spectrometry for the Confirmation of Caribbean Ciguatoxin-1 as the Main Toxin Responsible for Ciguatera Poisoning Caused by Fish from European Atlantic Coasts
Source: Toxins (Basel). 2020 Apr 21;12(4):267. doi: 10.3390/toxins12040267 (PMC7232264; doi:10.3390/toxins12040267)
Supplement: Supplementary file 1 [file toxins-12-00267-s001.pdf]

# Supplementary materials: Liquid Chromatography Coupled to High Resolution Mass Spectrometry for the Confirmation of Caribbean Ciguatoxin-1 as the Main Toxin Responsible for Ciguatera Poisoning Caused by Fish from European Atlantic Coasts

**Pablo Estevez, Manoella Sibat, José Manuel Leão-Martins, Pedro Reis Costa, Ana Gago-Martínez and Philipp Hess**

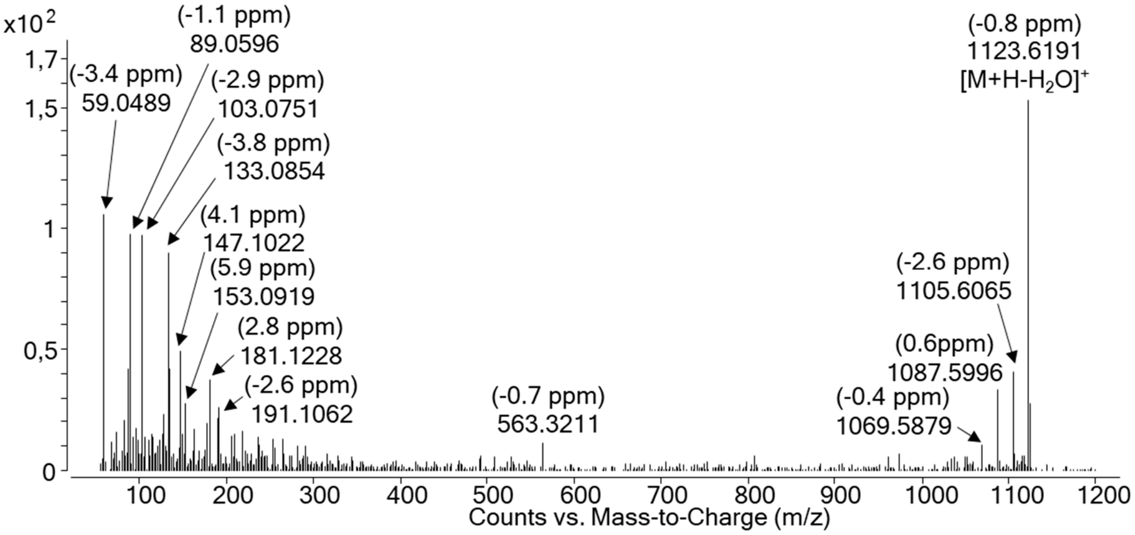


**Figure S1.** ESI+ Targeted MS/MS spectra of C-CTX1 LRM (123 ng/mL approx.) selecting C-CTX1 m/z 1123.6200 [M+H-$H_2O$]+ at an average collision energy of 20, 40 and 60 eV.

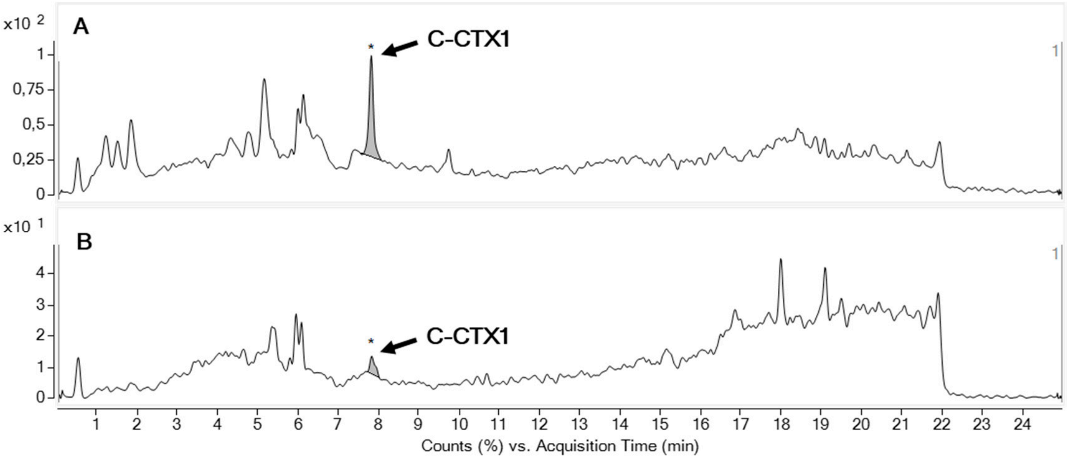


**Figure S2.** LC-HRMS chromatogram of **A**) C-CTX1 LRM (123 ng/mL approx.) at 7.8 min; **B**) C-CTX1 detected at 7.8 min in a naturally contaminated sample of barred hogfish (*Bodianus scrofa)* from Selvagens Islands (Portugal)

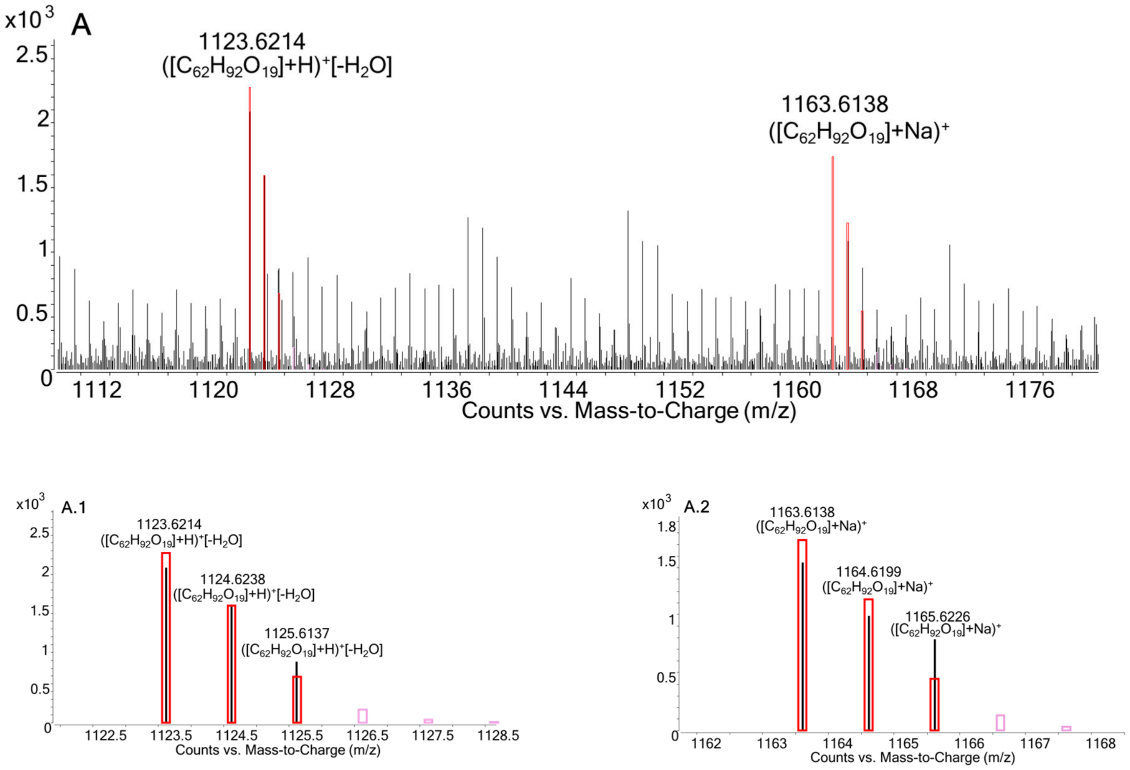


**Figure S3. A**) ESI⁺ Full scan MS spectra of Barred hogfish (*Bodianus scrofa)* from Selvagens Islands (Portugal); **A.1**) Zoom of C-CTX1 *m/z* 1123.6214 [M+H-$H_2O$]⁺; **A.2**) Zoom of C-CTX1 *m/z* 1163.6138 [M+Na]⁺.

**Table S1.** Accurate mono-isotopic masses (theoretical and measured) of C-CTX1 after ESI⁺ Targeted MS/MS analysis selecting [M+H-($H_2O$)]⁺ at *m/z* 1123.6200 with an averaged collision energy of 20, 40 and 60 eV (**A**) and 30, 50 and 70 eV (**B**).

| Ion | Molecular Formula | Theoretical *m/z* | A | | B | |
|---|---|---|---|---|---|---|
| | | | Measured *m/z* | Error (ppm) | Measured *m/z* | Error (ppm) |
| [M+H-$H_2O$]⁺ | $C_{62}H_{91}O_{18}^+$ | 1123.6200 | 1123.6191 | −0.8 | 1123.6158 | −3.7 |
| [M+H−2$H_2O$]⁺ | $C_{62}H_{89}O_{17}^+$ | 1105.6094 | 1105.6065 | −2.6 | 1105.6063 | −2.8 |
| [M+H−3$H_2O$]⁺ | $C_{62}H_{87}O_{16}^+$ | 1087.5989 | 1087.5996 | 0.6 | 1087.5933 | −5.1 |
| [M+H−4$H_2O$]⁺ | $C_{62}H_{85}O_{15}^+$ | 1069.5883 | 1069.5879 | −0.4 | 1069.5865 | −1.7 |
| | $C_{31}H_{47}O_9^+$ | 563.3215 | 563.3211 | −0.7 | n.d. | n.d. |
| | $C_{31}H_{45}O_8^+$ | 545.3109 | n.d. | n.d. | 545.3072 | −6.8 |
| | $C_{31}H_{43}O_7^+$ | 527.3003 | n.d. | n.d. | 527.2953 | −9.5 |
| | $C_{31}H_{41}O_6^+$ | 509.2898 | n.d. | n.d. | 509.2873 | −4.9 |
| | $C_{14}H_{21}O_4^+$ | 253.1434 | n.d. | n.d. | 253.1431 | −1.2 |
| | $C_{14}H_{19}O_3^+$ | 235.1329 | n.d. | n.d. | 235.1334 | 2.1 |
| | $C_{12}H_{17}O_3^+$ | 209.1172 | n.d. | n.d. | 209.1173 | 0.5 |
| | $C_{12}H_{15}O_2^+$ | 191.1067 | 191.1062 | −2.6 | 191.1066 | −0.5 |
| | $C_{11}H_{17}O_2^+$ | 181.1223 | 181.1228 | 2.8 | 181.1224 | 0.6 |
| | $C_9H_{13}O_2^+$ | 153.0910 | 153.0919 | 5.9 | n.d. | n.d. |
| | $C_7H_{15}O_3^+$ | 147.1016 | 147.1022 | 4.1 | 147.1011 | −3.4 |
| | $C_6H_{13}O_3^+$ | 133.0859 | 133.0854 | −3.8 | 133.0856 | −2.3 |
| | $C_5H_{11}O_2^+$ | 103.0754 | 103.0751 | −2.9 | 103.0751 | −2.9 |
| | $C_4H_9O_2^+$ | 89.0597 | 89.0596 | −1.1 | 89.0595 | −2.2 |
| | $C_3H_7O^+$ | 59.0491 | 59.0489 | −3.4 | 59.0487 | −6.8 |
